# Delayed diagnosis of pemphigus herpetiformis in a patient with dupilumab-associated hypereosinophilia

**DOI:** 10.1016/j.jdcr.2025.06.003

**Published:** 2025-06-18

**Authors:** Sarah E. Smith, Leah A. Swanson, Alex Wonnaparhown, David J. DiCaudo, Steven A. Nelson, Michael Kasperkiewicz

**Affiliations:** a Department of Dermatology, Mayo Clinic, Scottsdale, Arizona; b Department of Laboratory Medicine and Pathology, Mayo Clinic, Scottsdale, Arizona; c Department of Allergy and Immunology, Mayo Clinic, Scottsdale, Arizona; d Division of Dermatology, Department of Medicine, David Geffen School of Medicine at University of California Los Angeles, Los Angeles, California

**Keywords:** atopic dermatitis, autoimmune blistering disorder, dupilumab, eosinophilia, immunobullous disease, pemphigus herpetiformis

## Introduction

Pemphigus herpetiformis (PH) is an uncommon form of the pemphigus family mediated by pathogenic autoantibodies directed against desmosomal adhesion proteins, first introduced by Jabłońska et al in 1975.^1^ Its diagnosis is challenging since it is a rare immunobullous disease variant with unusual presentation that can mimic several other pruritic inflammatory dermatoses including atopic dermatitis (AD) and bullous pemphigoid (BP).^1^ PH and AD represent prototypes of the epidermal group of a spectrum of eosinophilic dermatoses, whereas BP is a prototypic eosinophilic dermatosis of the dermal-epidermal junction.^2^ Dupilumab is a human monoclonal antibody that binds to the shared interleukin (IL) 4 receptor alpha chain, which leads to inhibition of both IL-4 and IL-13 signaling and has been shown to be effective in treating different eosinophilic dermatoses including AD and BP.^3^ Herein, we describe a patient on long-term, unsuccessful treatment with dupilumab for suspected AD who developed hypereosinophilia which resolved following discontinuation of this drug after being finally diagnosed with PH. We conclude with a discussion on potential misdiagnosis and/or triggering of PH by dupilumab.

## Case report

**Fig 1:**
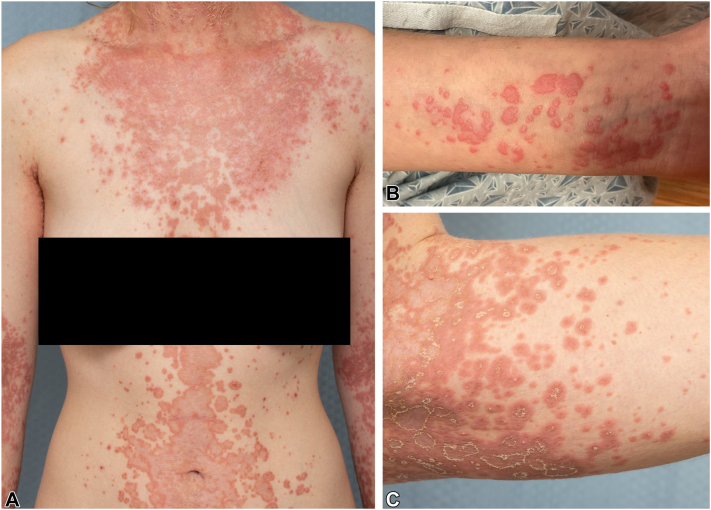
**A-C,** Clinical photographs of the neck, trunk, and extremities.

**Fig 2:**
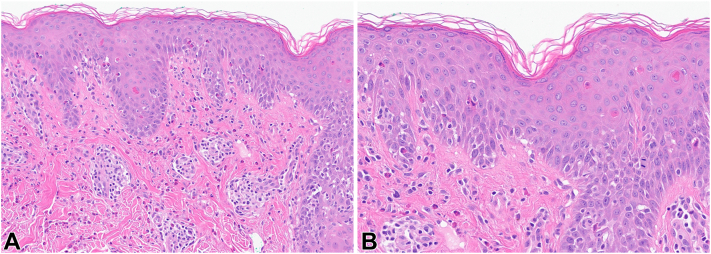
**A** and **B,** Skin biopsy from the neck (hematoxylin and eosin stain); original magnification ×200 **(A)** and ×400 **(B)**.

A woman in her 20s presented with a 6-year history of a widespread pruritic rash. History was significant for allergic rhinitis, anxiety, depression, attention deficit hyperactivity disorder, irritable bowel syndrome, and migraine. Family history was significant for AD. Failed treatments for suspected AD included topical corticosteroids, topical calcineurin inhibitors, topical phosphodiesterase-4 inhibitor, topical janus-activated kinase 1 and 2 inhibitor, and narrowband ultraviolet B phototherapy. At the time of presentation, she was on dupilumab (300 mg every other week initially, later increased to weekly without benefit) which was started about 4 months after rash onset. Oral prednisone was effective in clearing the rash at doses of > 0.5 mg/kg, but it usually reoccurred once the dose was decreased. Examination revealed itching, widespread, grouped, coalescent, erythematous, lichenified, and partly scaly plaques and papules in an annular-shaped pattern. No blisters or mucosal involvement were observed (Fig 1). Punch biopsies demonstrated prominent eosinophilic spongiosis and scattered intraepidermal apoptotic keratinocytes. The dermal infiltrate consisted of many eosinophils, lymphocytes, and focal neutrophils (Fig 2). Direct immunofluorescence revealed weak cell surface immunoglobulin G (IgG) and C3 as well as weak granular basement membrane C3 staining. Indirect immunofluorescence showed cell surface IgG (1:640) and immunoglobulin A (1:80) antibodies on primate esophagus. There were no IgG antibodies detected by immunofluorescence testing on rat bladder. By enzyme-linked immunosorbent assay, antidesmoglein 1 antibody was elevated at 47 U/mL (neg < 14), antidesmoglein 3 antibody at 17 U/mL (neg < 9), and anti-BP180 antibody at 19 U/mL (neg < 14), whereas other serologies (anti-BP230, antilaminin 332, anti-p200, anti-type VII collagen, and antinuclear antibodies/extractable nuclear antigen antibodies) were negative. Laboratories were otherwise remarkable for marked eosinophilia of 2430/mm^3^ that increased to 4210/mm^3^ (nl 30-480/mm^3^) checked 8 days apart. A workup for Coccidioides, Toxocara, Amebiasis, and Stronglyoides was negative. Results of whole body positron emission tomography and computed tomography scan as well as transabdominal/transvaginal ultrasound were also unremarkable.

Her clinical presentation, histopathology, and serologic studies were most compatible with PH. In particular, our patient met 2 previously proposed mandatory criteria for the diagnosis of PH (grouped/herpetiform distribution of itching erythematous vesicular, bullous, or papular lesions, often in an annular-shaped pattern as well as intercellular IgG and/or C3 in epithelium).^1^ Dupilumab was discontinued due to suspected drug-induced eosinophilia and concern that it triggered her underlying PH. Treatment with prednisone (up to 1 mg/kg), dapsone (50-200 mg daily), and rituximab (1 g each 2 weeks apart) was performed. Eosinophilia resolved quickly after discontinuation of dupilumab, and her symptoms overall improved along with a decline of autoantibodies except for minor flare-ups mostly localized to the eyelids.

## Discussion

Although dupilumab is generally well tolerated, eosinophilia due to inhibition of IL-4-/IL-13–mediated chemokines and adhesion molecules that assist with eosinophil migration from blood into tissue has been observed.^4^ The molecular pathophysiology and direct association between dupilumab and development of hypereosinophilic syndrome are not entirely clear at this time. Organs that may be affected in this syndrome include the lungs (severe asthma, infiltrative pulmonary disease, pleural effusions, pulmonary embolism, or fibrosis), heart (myocarditis, myocardial necrosis, endocardial damage, heart failure, and thromboembolic complications), nervous system (ischemic attacks, peripheral neuropathies, and encephalopathy), and, to a lesser extent, gastrointestinal tract, kidney, and skin (erythema, angioedema, ulceration, pruritus, and eczema).^5^

Similar to AD and BP, PH is also considered a Th2-related disease in which eosinophils play a major pathophysiologic role. IL-5, IL-8, IL-31, and thymus and activation-regulated chemokine have been proposed to be involved in the autoantibody-mediated inflammatory response resulting in eosinophilic spongiosis with or without acantholysis in PH.^1^^,^^6^ To our knowledge, this is the first reported case of potential triggering of PH by dupilumab, but this link remains unproven, particularly considering that the clinical improvement of our patient seen after withdrawal of this drug may have been primarily attributed to concurrent immunosuppressive treatment. Similarly, dupilumab-treated patients with AD experiencing worsening of their disease have been reported, although the underlying mechanism of this paradoxical reaction is poorly understood.^7^ It may be assumed that PH was either initially misdiagnosed as AD or AD transitioned to PH during the course of the disease and dupilumab treatment.

Our case highlights the need for considering PH as an important differential diagnosis of more common pruritic dermatoses like AD and BP, particularly in patients with a treatment-refractory course. In fact, patients with AD and BP, especially its nonbullous form, may not only show a clinical but also a histological overlap (eosinophilic spongiosis) with PH and must therefore be differentiated by appropriate immunological testing of circulating and tissue-bound autoantibodies.^1^^,^^2^ Also, patients who lack improvement with dupilumab should be evaluated for hypereosinophilia. More basic and clinical research is needed to elucidate the relationship between blockade of the IL-4/IL-13 pathway, hypereosinophilia, and lack of improvement or even possible triggering of eosinophilic dermatoses like PH by dupilumab.

## Conflicts of interest

None disclosed.
